# Core and conditionally rare taxa as indicators of agricultural drainage ditch and stream health and function

**DOI:** 10.1186/s12866-023-02755-7

**Published:** 2023-03-07

**Authors:** Yichao Shi, Izhar U. H. Khan, Devon Radford, Galen Guo, Mark Sunohara, Emilia Craiovan, David R. Lapen, Phillip Pham, Wen Chen

**Affiliations:** 1 Ottawa Research and Development Centre, Agriculture and Agri-Food Canada, 960 Carling Avenue, Ottawa, Canada; 2 Department of Biology, University of Ottawa, Marie-Curie Private, Ottawa, ON K1N 9A7 Canada

**Keywords:** Agricultural watersheds, Core microbiome, Conditionally rare taxa (CRT), Ecosystem stability, Environmental risk indicators, Freshwater microbiome

## Abstract

**Background:**

The freshwater microbiome regulates aquatic ecological functionality, nutrient cycling, pathogenicity, and has the capacity to dissipate and regulate pollutants. Agricultural drainage ditches are ubiquitous in regions where field drainage is necessary for crop productivity, and as such, are first-line receptors of agricultural drainage and runoff. How bacterial communities in these systems respond to environmental and anthropogenic stressors are not well understood. In this study, we carried out a three year study in an agriculturally dominated river basin in eastern Ontario, Canada to explore the spatial and temporal dynamics of the core and conditionally rare taxa (CRT) of the instream bacterial communities using a 16S rRNA gene amplicon sequencing approach. Water samples were collected from nine stream and drainage ditch sites that represented the influence of a range of upstream land uses.

**Results:**

The cross-site core and CRT accounted for 5.6% of the total number of amplicon sequence variants (ASVs), yet represented, on average, over 60% of the heterogeneity of the overall bacterial community; hence, well reflected the spatial and temporal microbial dynamics in the water courses. The contribution of core microbiome to the overall community heterogeneity represented the community stability across all sampling sites. CRT was primarily composed of functional taxa involved in nitrogen (N) cycling and was linked to nutrient loading, water levels, and flow, particularly in the smaller agricultural drainage ditches. Both the core and the CRT were sensitive responders to changes in hydrological conditions.

**Conclusions:**

We demonstrate that core and CRT can be considered as holistic tools to explore the temporal and spatial variations of the aquatic microbial community and can be used as sensitive indicators of the health and function of agriculturally dominated water courses. This approach also reduces computational complexity in relation to analyzing the entire microbial community for such purposes.

**Supplementary Information:**

The online version contains supplementary material available at 10.1186/s12866-023-02755-7.

## Introduction

Agriculture can impact a waterway’s ability to support, regulate, and sustain ecosystem services and biological functionality [1, 2]. If agriculture drainage and runoff are not managed properly, water contamination [3] and associated biodiversity loss can occur [4]. Moreover, climate and land use changes could further perturb these influences [5, 6]; but such changes may also have unexpected positive feedbacks [6].

Agricultural drainage ditches are typically narrow incised, manmade, linear waterways that are ubiquitous in agricultural regions where surface runoff channeling and artificial subsurface drainage (tile drainage) are necessary to drain fields for agricultural productivity [7]. Drainage ditch networks can occupy thousands of kilometers in river basins, and represent some of the only ‘semi-naturalized’ aquatic ecosystems in otherwise depauperate field-scapes [8]. Furthermore, agricultural drainage ditches are direct receivers of water influenced by agriculture activities; therefore, they play critical roles in regulating water flow, supporting wildlife habitat, governing greenhouse gas emissions [9, 10], as well as assimilating and dissipating agro-chemicals and pathogens [11, 12]. The sustainable management of agricultural drainage ditches can help to support biodiversity and reduce public health and environmental impacts.

Microorganisms play key roles in ecosystem function and services, including primary productivity, greenhouse gas emission, carbon (C) sequestration, and biogeochemical cycling of nutrients and contaminants [13, 14]. Yet, environmental and anthropogenic stressors can drive and control such processes [15]. Previous studies have highlighted that bacterial communities in freshwater ecosystems are sensitive to both short term and gradual changes in surface water conditions [16, 17]. Some drivers that determine the composition and function of the aquatic microbiome in agriculturally-dominated watersheds include adjacent agricultural land uses (i.e., nature of agro-chemical applications, tillage practices, soil condition), water quality, stream velocity, connectivity, and water depth, surface and subsurface water inputs from adjacent fields, and resident aquatic biota and substrate [18–20]. For instance, higher nitrate and phosphorus (P) content and turbidity of surface water under the influence of agricultural activities can lead to the enrichment of *Proteobacteria* and *Bacteroidetes*, which comprise important functional guilds like denitrifiers and fermenters [21] as well as the surge of fecal indicator bacteria [22].

Notwithstanding the foundational importance of the bacterial microbiome in any aquatic ecosystem, studies have not fully addressed how bacterial communities (and functions) in agricultural drainage ditches (which often reflect true source waters in many agriculturally dominated watersheds) respond to flow and water depth changes (e.g., intermittency to high flow and vice versa), routine management and maintenance of ditch integrity, and direct inputs of agro-chemicals, sediments, and bacteria from agricultural activities. Such studies would help us better understand how, when, and where the bacterial microbiome in these ditches respond to different anthropogenic/environmental stressors, as well as the degree to which associated ecosystem service regulating and supporting functions are subsequently affected. For example, Chen et al. [20] demonstrated a seasonal diversity shift of aquatic microbiomes and found that nitrifiers (e.g., *Nitrospiraceae* spp.) were more abundant in agricultural streams than larger surface water courses with more vegetated upstream and adjacent land uses. García-Armisen et al. [23] and Xu et al. [24] have also shown seasonal changes in stream bacterial community composition which reflected upstream wastewater discharge, while Flynn et al. [25] attributed the source of *Bacterioidales* bacteria (ruminant-specific vs. human-specific) in surface water to on- and off-season outdoor grazing of livestock. Previous studies have primarily focused on qualifying changes in bacterial diversity and compositional and functional features of the aquatic microbiome, but few studies have directly explored bacterial communities in agricultural drainage ditch water as indicators of ditch aquatic ecosystem health and stress. Bacterial community-based indices of biotic integrity [26] could be used for such assessments [27].

To define the key features of the aquatic microbiome to be effective and robust indicators of aquatic ecosystem stress and function, it is necessary to quantify bacterial community changes as a result of abrupt environmental stability shifts that are typically characteristic of many smaller agricultural water courses. Shade et al. [28–30] proposed to deterministically explore the abundance-occupancy distributions of microbial community members from a broad range of ecosystems and to systematically characterize the “core microbiome” and “conditionally rare taxa” (CRT). A core subcommunity is made up of taxa that are consistently observed in high abundance over time or space [30], whereas CRT stay at or below the detection limit but sporadically boost to appreciable abundance as a result of changes in environmental conditions [29]. Both the dominant and rare bacterial taxa may play diverse roles and can reside in different ecological niches [31], thereby providing greater insight into microbiome resiliency and functional redundancy. Hence, defining the core and CRT subcommunities and evaluating their collective contributions to the variance in microbiome compositional structure, could help quantify community stability and resilience [32] in the dynamic drainage ditch environments that act as first-line receptors of agriculturally impacted effluent.

The present study integrated land use, hydrological, weather, and water physicochemical information to determine environmental drivers influencing the seasonal variability of aquatic microbial communities in a suite of agricultural drainage ditches and streams situated in an agriculturally-dominated river basin in Eastern Ontario, Canada. The freshwater bacterial communities were characterized through metabarcoding the V4-V5 regions of the 16S rRNA gene. We hypothesized that 1) the core taxa and the CRT can represent and reflect the spatial and temporal variation of the whole aquatic bacterial community; 2) the aquatic bacterial communities within agricultural drainage ditches have lower stability compared to larger and/or more diluted water ways; and 3) specific core and CRT are functional guilds critical to the health and function of agricultural ditches and streams.

## Materials and methods

### Study sites

**Fig. 1:**
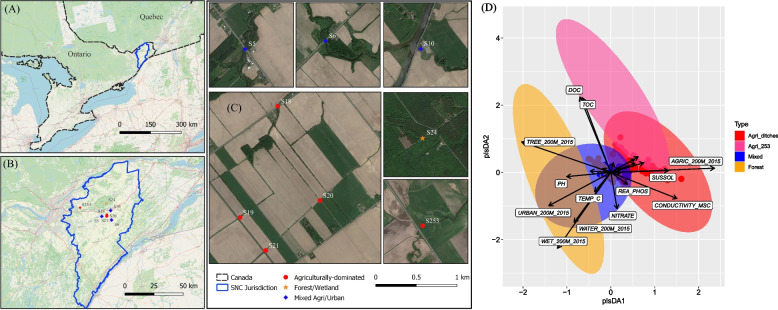
Study sites. Location of South Nation river basin in eastern Ontario (**A**); Sample locations in the river basin (**B**); (**C**) Land use features of the nine stream sites: S18, S19, S20, and S21 are agricultural drainage ditch sites, S253 (denoted as Agri_253) is located on a larger stream (Strahler stream order = 4), S5, S6, and S10 are under mixed agricultural and urban development, while S24 is in a forested area under no known influence of anthropogenic activity. Maps sources: Google Maps, OpenStreetMap, and Conservation Ontario (https://camaps.maps.arcgis.com/home/index.html); (**D**) Partial least squares discriminant analysis (PLS-DA) for stream site classification using land use, hydrological features, and water physiochemical properties. The detailed descriptions of the water physicochemical properties can be found in Table 2

This study was conducted in the agriculturally dominated South Nation River basin located in Eastern Ontario, Canada. The basin has a catchment area of approximately 3900 km^2^ (Fig. 1A). Six water sampling sites were selected in 2016 (Site: S5, S6, S10, S18, S20, and S24) and an additional three were added (S19, S21, S253) in 2017 and 2018 (Fig. 1, Table 1). These study sites have been described in detail previously [11, 20]. Dairy livestock farming is a dominant land use in the basin, and liquid and solid livestock manure is frequently applied to land as fertilizer in spring and fall. Corn, soybeans, and grass or alfalfa forage are the predominant crops grown.

**Table 1:** Sample site descriptions. Land uses defined for 200 m radius around each sample site

SITE ID	Land use type	Upstream contributing area (km^2^)	Strahler stream order	Water (% land)	Urban/Developed (% land)	Forest (% land)	Wetland (% land)	Agriculture (% land)	Other (% land)
S5	Mixed	81	4	0	7.79	71.46	1.93	16.53	2.29
S6	Mixed	176	5	0	8.42	31.14	0	60.44	0
S10	Mixed	67.5	4	23.77	10.34	2.91	3.63	57.33	2.01
S18	Agriculture	< 5	2	0	0.63	0	0	99.37	0
S19	Agriculture	< 5	2	0	3.7	0	0	96.3	0
S20	Agriculture	< 5	2	0	0	0	0	100	0
S21	Agriculture	< 5	2	0	13.33	0	0	86.67	0
S253	Agriculture	51	4	0	7.57	0	0	92.43	0
S24	Forest	< 5	1	0	6.31	93.69	0	0	0

Sampling sites are located on surface water catchments ranging from < 5 to ∼180 km^2^ (Table 1). S18, S19, S20 and S21 are located on agricultural drainage ditches (Strahler stream orders ≤2) fed almost exclusively by agricultural sub-surface tile drainage [33]. S253 (denoted as Agri_253) is located on a larger stream (Strahler stream order 4) fed primarily by agriculturally impacted waters. S5, S6, and S10 are intermediate tributaries (Strahler stream order 4–5) that feed the main river directly and are influenced by a mix of agricultural and urban activities. S24 is located on a small stream (Strahler stream order 1). It drains a forested-wetland area not impacted by any known anthropogenic land use activity. S24 serves as a proxy reference site in the context of anthropogenic land use activity in the study [12]. Only one reference site was included in our study due to accessibility and availability in a region where most watersheds are impacted in some way by anthropogenic activity. We sampled all sites that were logistically possible within the same day in order to process the samples within 24 hours.

Land use upstream of each site was characterized using the methods described by Wilkes et al. [11] and was classified coarsely into agricultural land, urban/developed land, treed land, wetlands, water, or other (Table 1). Land use data were obtained in the form of raster layers from Agriculture and Agri-Food Canada (AAFC)‘s Annual Crop Inventory [34]. Surface water catchment areas (entire catchment upstream of sample site, and catchment area associated with maximum stream length of 5 km upstream of a sample site) and flow direction were determined within ArcMap 9.2 (Environmental Systems Research Institute, Redlands, CA). Stream order was determined using methods described by Lyautey et al. [35]. Daily air temperature and rainfall were measured from a Hobo weather station (Onset Computer Corporation, Bourne, MA) near S20. Cumulative rainfall was calculated for the day of sampling and 1, 2, 3, 5, 7, and 10 days prior to sampling.

### Water sample collection, processing, and DNA extraction

Water samples were collected 0–50 cm below the surface on a bi-weekly basis from April to November in 2016 to 2018 using protocols described previously [20]. In brief, water samples were collected in 2 L sterile bottles, immediately placed on ice, and returned to AAFC’s Ottawa laboratories and processed within 24 h of collection for microbiological analyses. The physiochemical properties of the freshly collected water samples (Table 2), including pH, dissolved oxygen, specific conductivity, turbidity, oxidation-reduction potential, and concentrations of ammonia and ammonium, nitrite and nitrate, total Kjeldahl N, total and reactive P, were measured using methods described by Wilkes et al. [11]. Measurement of total suspended solid, dissolved organic C, and total organic C was based on APHA 2540D methods as described by Rice et al. [36].

**Table 2:** Land use and water physio-chemical variables used for data analysis in this study

Variable name	Variable description (unit)
PH	pH read with the YSI mini sonde in water
CONDUCTIVITY_MSC	Water specific conductivity in millisiemens per centimeter read with the YSI mini sonde in water (mS cm^−1^)
DISS.OXYGEN_MGL	Dissolved oxygen in milligrams per liter read with the YSI mini sonde in water (mg L^− 1^)
ORP_MV	Oxidation reduction potential of sample water (mV)
TURBIDITY_NTU	Cloudiness of sample water as measured with a nephelometer sensor (NTU; nephelometric turbidity units)
AMIA_AMN	Ammonia + ammonium concentrations in (mg L^−1^)
NITRITE	Nitrite concentrations (mg L^− 1^)
NITRATE	Nitrate concentrations (mg L^− 1^)
REA_PHOS	Reactive phosphorus concentrations (mg L^− 1^)
TOTKN	Total Kjeldahl nitrogen (mg L^− 1^)
TOTPHO	Total phosphorus concentrations (mg L^− 1^)
SUSSOL	Total Suspended Solids (mg L^− 1^)
DOC	Dissolved organic carbon (mg L^− 1^)
TOC	Total organic carbon (mg L^− 1^)
RU_DISM3S	Discharge of Castor River at Russell station (02LB006), daily mean in cubic meter per second (m^3^ s^−1^)
BE_DISM3S	Discharge of Payne River near Berwick station (02LB022), daily mean in cubic meter per second (m^3^ s^−1^)
DAILY_AVG_SOLAR_RADIATION_W.m^2^	Daily average of solar radiation (W m^−2^)
(land use)_200M_2015	Proportion of land use types immediately surrounding sample site (200 m radius of site); agriculture (AGRIC), urban/developed (URBAN), tree/forest (TREE), wetland (WET), and water (WATER) (%)
Totalrain_xd	Total rainfall cumulated at the day of sampling (x = 1) and x (2, 3, 5, 7, 10) days in advance of sampling day (mm)
Avg_temp_C_xd	Daily mean air temperature for day of sampling and x = 1, 2, 3, 5, 7, 10 days in advance of sampling day (°C)

For enhanced recovery of microbial community and diversity and to avoid clogging of filters with smaller pore size, a two-tier water filtration approach was applied where 500 mL of water was first filtered through 0.7 μm borosilicate glass filters (Thermo Fisher, Ottawa, ON, Canada), then the filtrate filtered through 0.22 μm sterile nitrocellulose filters (Millipore, Billerica, MA, USA). Each filter (0.7 and 0.22 μm) was subjected to the total nucleic acid extraction using DNeasy PowerSoil Kit (Qiagen, U.S.A) following the manufacturer’s instructions. The quantity and quality of DNA was evaluated by Qubit 3.0 fluorometer and 1% agarose gel electrophoresis with 1x TAE buffer (0.04 M Tris-acetate, 0.001 M EDTA, pH 7.8). All DNA samples were stored at − 80 °C until further use.

### Sequencing library preparation, high-throughput sequencing, and raw metabarcoding data processing

Amplicon-based sequencing libraries were prepared as follows: PCR amplification was carried out using Qiagen HotStar MasterMix (Toronto, ON, Canada), 16S rRNA (V4-V5 hyper-variable region) gene-specific primer pair 515F-Y (5′-GTG YCA GCM GCC GCG GTA A-3′) and 926R (5′-CCG YCA ATT YMT TTR AGT TT-3′) and DNA template [37] with an initial denaturation at 95 °C for 3 min followed by 25 cycles of denaturation at 95 °C for 30 s, annealing temperature of 55 °C for 30 s, extension at 72 °C for 30 s and final extension at 72 °C for 5 min. Each amplicon was purified using NucleoMag NGS Clean-up and Size Select beads. The libraries were pooled in equimolar ratios and then diluted pools were prepared for sequencing according to the MiSeq System Denature and Dilute Libraries Guide. The sequencing libraries were loaded on an Illumina MiSeq sequencer and sequenced using a 500-cycle MiSeq Reagent Kit v2 (San Diego, CA, USA) which generated 2 × 250 bp reads. DNA samples were store at –80 °C before and after sequencing library preparation.

The paired-end raw reads were processed using DADA2 (1.14) for denoising, chimera detection, and the amplicon sequence variants (ASVs) inference [38]. Filtering was performed using default parameters. The raw forward and reverse reads were truncated at 240 nt. The taxonomic assignment was performed by training the Naive Bayes classifier q2-feature-classifier [39] using Greengenes reference database (version 13_8) with a minimum bootstrap confidence at 80%. A total of 26,867,767 high-quality reads were retained in the final ASV abundance table, with 34,534 ± 17,691 (MEAN ± SD) reads per sample. Functional annotation of the ASV table was inferred using PICRUSt 2 [40].

### Statistical analysis

All statistical analysis were performed in R (v.4.2.0) [41] unless stated otherwise. ASVs obtained from the sequencing data of DNA extracted from 0.22 and 0.7 μm filters for each sample were combined and then rarefied to 13,430 reads per sample for a total of 316 samples. In this observational ecology study, all sampling sites are situated on different watersheds and geographically distant (S5, S6, S10, S24, S253, ranging from 7.3 to 20.2 km apart). S18 and S19 (1.45 km apart) however, are on the same watershed and S18 is downstream of S19. S20 and S21 (~ 1 km apart) are on an adjacent watershed and S20 is downstream of S21. Because streams on different watersheds are receiving almost exclusively the water associated with their respective watershed catchments, the forested reference (S24) and mixed-use (S5, S6, and S10) sites that are on separate catchment areas were treated as formalized replicates for statistical purposes. We treated S18 + S19 and S20 + S21 as blocks and hence the sample size of the stream sites (referred to as blocks) was seven (*n* = 7, S5, S6, S10, S18 + S19, S20 + S21, S253, and S24). Linear mixed-effects models were used to assess the effects of land use types on water physiochemical properties, the abundance of taxa at different taxonomic ranks, and the N function groups using *lme* function in the *nlme* package [42] at a significance level of *P* ≤ 0.05. The land use type was treated as a fixed effect, and block and sampling date were treated as random effects; sampling date was treated as a repeated measure. Pairwise comparison was conducted using *posthoc_pairwise* function in *grafify* package with “tukey” adjustment [43]. Partial least squares discriminant analysis (PLS-DA, *DiscriMiner* package) [44] was used to discriminate SNR sampling sites based on independent metadata data.

To characterize the temporal stability of the microbial communities, we identified the core taxa and CRT for all sampling sites and for agricultural drainage ditch sites only using methods proposed by Shade and Stopnisek [30] and Shade et al. [29]. In brief, core ASVs were identified based on their abundance-occupancy distributions. All ASVs were first ranked based on their abundance and presence or absence over time, with ASVs being both more abundant and prevalent ranked higher. The core ASVs were selected by sequentially including next-ranked ASVs and then calculating their contributions to the overall community heterogeneity over time. This was done by dividing the Bray-Curtis similarity of the core ASVs by that of the whole community (i.e., all ASVs). As higher-ranked ASVs being consecutively included in the core microbiome, we observed higher percent of the beta-diversity being explained by the core community. The selection process ended when the inclusion of the last ranked ASVs only explained an additional 0.5% or less of the beta-diversity of the whole community. To identify the CRT, we examined the frequency of the ASV abundance (≥ 0.1%) over time for a bimodal distribution. The coefficient of bimodality, b, was set at 0.90 [29].

The percent contribution of the core taxa and CRT to the overall community beta-diversity was represented by dividing the Bray-Curtis dissimilarity of core taxa or CRT by that of the overall community at each sampling time point. We did not select the core taxa and CRT for the forested site (S24) and the site located on a larger agricultural stream (S253), because we were only able to access a single site in these landscapes. Consequently, the Bray-Curtis dissimilarity at each sampling time point could not be calculated for these two sites. The impact of land use class (i.e., agricultural drainage ditch sites vs. mixed-use sites) on the contributions of core taxa and CRT was evaluated by performing linear mixed-effects modeling with land use class as a fixed factor, and sampling date as a repeated measure. The *community stability* [45] was calculated for the combined core and CRT by dividing the summed species abundances by the corresponding standard deviations over successive time intervals using the *community_stability* function in the *codyn* package [46]. Larger values of *community stability* represent greater temporal stability of the freshwater bacterial community. The effect of land use class on the stability was evaluated by performing linear mixed-effects modeling with land use type as a fixed factor, block as a random factor, and sampling year as repeated measures.

The importance of environmental factors influencing the temporal variation of the core and CRT contribution to the overall community beta-diversity were ranked using the *randomForest* function in the randomForest package (version 4.6–14) [47]. To select sensitive microbial indicators for agricultural drainage ditch sites, their core subcommunity was screened for ASVs that were significantly more abundant at agricultural drainage ditch sites than those at mixed-use or forested sites. The selected ASVs of the same genus were aggregated in abundance; their relationships with environmental parameters were evaluated by Spearman’s rank-order correlation analysis using the *rcorr* function in the Hmisc package [48]. PICRUSt 2 [40] was used to predict ASVs affiliated with KEGG orthologs (KO) and pathways. The seasonal dynamics of CRT affiliated with N metabolism were presented by *stat_smooth* function in ggplot2 using loess smoothing method [49].

## Results

### Freshwater physiochemical properties and land use classes

The PLS-DA (Fig. 1D) showed that most water physiochemical variables, except for oxidation-reduction potential, were significantly (*P* < 0.05) associated with land use class (Table 3). The water in smaller agricultural drainage ditches had significantly higher conductivity and total suspended solids across all sampling seasons, relative to other sites (Table 3, supplementary Fig. S1). Transient water quality shifts were observed in 2016 and 2018, where surges of turbidity, total suspended solids, N, and P were detected at agricultural drainage ditches, but not at other sites (supplementary Fig. S1). In contrast, the forested reference site (S24) had higher organic C (stream drains from a treed wetland), and lower turbidity, N, and P, relative to agricultural drainage ditches (*alpha* < 0.05) (Table 3, supplementary Fig. S1).

**Table 3:** Statistical analysis of water physicochemical properties under different land use classes (2016-18)

Parameters^a^	Agri_ditches^b^				Agri_253^c^				Forest				Mixed				*P* value^d^
	N	Mean	Min	Max	N	Mean	Min	Max	N	Mean	Min	Max	N	Mean	Min	Max	
TEMP_C	122	14.75	2.57	26.06	27	15.31	3.48	22.71	40	13.74	2.82	21.47	126	16.98	2.94	27.06	0.161
PH	122	7.74	7	8.43	27	7.64	6.53	8.65	40	8.03	7	9.19	126	7.92	7.12	8.5	0.099.
CONDUCTIVITY_MSC	122	0.82	0.004	1.981	27	0.76	0.357	2.511	40	0.19	0.035	1.246	126	0.62	0.232	1.72	0.007**^d^
DISS_OXYGEN_MGL	122	8.39	0.99	20.24	27	8.28	3.57	16.56	40	9.71	4.01	17.35	126	9.28	3.36	19.22	0.453
ORP_MV	122	249.60	−115.7	381.5	27	263.12	125.2	373.3	40	246.79	95	362	126	252.43	85.7	369.2	0.831
TURBIDITY_NTU	122	49.84	0.5	943.9	27	51.65	4	154.4	40	8.42	2.4	56.7	126	14.58	2.4	89.9	0.094.
AMIA_AMN	122	0.37	0.003	7.94	27	0.08	0.025	0.22	40	0.03	0.003	0.117	126	0.06	0.003	0.807	0.12
NITRITE	122	0.04	0.02	0.41	27	0.02	0.02	0.02	40	0.03	0.02	0.04	126	0.03	0.02	0.08	0.287
NITRATE	122	1.94	0.02	9.07	27	0.83	0.03	1.99	40	0.08	0.02	0.46	126	2.44	0.02	13.12	0.070.
REA_PHOS	122	0.04	0.004	0.343	27	0.03	0.012	0.062	40	0.01	0.004	0.02	126	0.03	0.004	0.201	0.797
TOTKN	122	1.48	0.38	12.2	27	1.56	0.88	3.05	40	0.74	0.27	1.46	126	0.85	0.47	2.05	0.070.
TOTPHO	122	0.18	0.009	1.47	27	0.19	0.072	1.037	40	0.04	0.015	0.098	126	0.09	0.013	0.301	0.205
SUSSOL	122	167.42	42	1420	27	93.63	28	350	40	18.53	4	88	126	72.15	34	141	0.009**
DOC	122	7.03	2.6	53.5	27	21.80	8.4	36.5	40	17.65	6.23	39.36	126	7.96	4.4	14.2	0.005**
TOC	122	7.33	2.64	58.79	27	24.54	8.44	44.67	40	18.38	6.28	40.42	126	8.30	4.8	14.99	0.004**

^a^ The detailed descriptions of the water physicochemical properties can be found in Table 2

^b^Agri_ditches, agricultural drainage ditch sites, including S18, S19, S20, and S21

^c^Agri_253, site S253 which is situated on a larger agriculturally-dominated stream

^d^ The *P* values represent the effects of land use classes based on linear mixed-effects modeling. Significance levels are denoted as **, *P* ≤ 0.01; ., *P* ≤ 0.1 is considered marginal significant

The seasonal variations in turbidity, ammonia and ammonium, total Kjeldahl N, and total P were significantly greater at agricultural drainage ditches (*P* < 0.05), where coefficients of variation were 2-fold or higher than those at other sites (supplementary Fig. S1). We observed higher dissolved oxygen and nitrate in water (especially at agricultural drainage ditch and mixed-use sites) and high relative water discharge in spring and fall, relative to summer months (supplementary Fig. S1). Compared with 2017, 2016 and 2018 were drier with the average rainfall from April 15 to November 15 being 372 mm in 2016 and 402 mm in 2018 (vs. 711 mm in 2017). In addition, the summers of 2016 and 2018 were hotter (air temperature in July averaged at 20.91 °C in 2016 and 22.5 °C in 2018 vs 19.8 °C in 2017), while the fall was cooler (8.8 °C in 2016 and 6.7 °C in 2018 vs 12.0 °C in 2017 in October) (supplementary Fig. S1).

### Core and conditionally rare taxa across all study sites represented overall community diversity dynamics

**Fig. 2:**
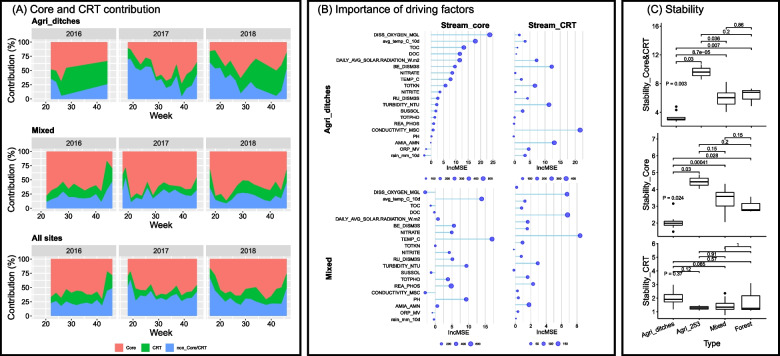
Dynamics of core and conditional rare taxa (CRT). (**A**) Area plots depict the percentage of overall Bray-Curtis dissimilarity (%) of aquatic microbiomes attributed to core and CRT dynamics over time in 2016–2018 at agricultural drainage ditch sites (top), mixed-use sites (middle), and all study sites (bottom). The red area represents the dynamics of core taxa contribution; the green area represents the dynamics of CRT contribution. The sampling started on May 24 in 2016, May 1 in 2017, and April 18 in 2018. Due to the drought conditions in 2016, only five samples were collected from agricultural drainage ditch sites. (**B**) The importance of environmental factors influencing the core (left) and CRT (right) contributions to overall beta-diversity at agricultural drainage ditch sites (top) and mixed-use sites (bottom) was ranked using random forest algorithm. The environmental factors shown in the y axis across four plots were ordered according to their importance ranking for Stream_core at agricultural drainage ditch sites. (**C**) Community stability in combined core and CRT (upper), core (middle), and CRT (lower) under four different land use classes. Agri_ditches, Agricultural drainage ditches; Agri_253, site S253 is situated on a larger agricultural stream (Strahler stream order 4). The detailed descriptions of the water physicochemical properties can be found in Table 2

We first identified the core microbiome (483 ASVs) and the CRT (6868 ASVs) across study sites, denoted as stream_core and stream_CRT, respectively (supplementary Fig. S2A). Collectively, the stream_core and stream_CRT accounted for 5.6% of the total number of ASVs of the overall stream bacterial community. Such a small number of ASVs, however, explained a large proportion of the Bray-Curtis dissimilarity of the whole aquatic bacterial community over time: averaging 68.8% (33.6–82.7%) for all sites, 62.3% (30.0–96.5%) for agricultural drainage ditch sites, and 72.7% (47.2–91.6%) for mixed-use sites (Fig. 2A). Of the remaining 124,458 ASVs that were not in stream_core or stream_CRT, 83% (103,579) ASVs had < 10 reads.

**Fig. 3:**
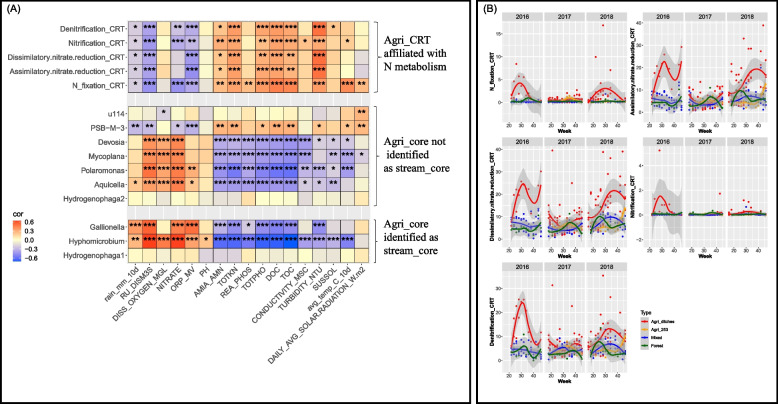
Functional guilds in the core taxa (agri_core) and CRT (agri_CRT) of agricultural drainage ditch sites. (**A**) Spearman’s rank correlation coefficient between the abundance of agri_core ASVs (represented in genus names) and environmental factors. The significance levels are denoted by *, *P* ≤ 0.05; **, *P* ≤ 0.01; ***, *P* ≤ 0.001; ****, *P* ≤ 0.0001. These agri_core ASVs were significantly more abundant at agricultural drainage ditch sites than at other stream sites. (**B**) Seasonal changes in CRT associated with N fixation (256 ASVs), assimilatory nitrate reduction (1562 ASVs), dissimilatory nitrate reduction (1978 ASVs), nitrification (35 ASVs), and denitrification (1243 ASVs) at agricultural drainage ditch sites using the “loess” smooth method. These functional guilds were more abundant at agricultural ditch sites than at other sampling sites. Agri_ditches, include S18, S19, S20, and S21; Agri_253, site S253 is situated on a larger agricultural stream (Strahler stream order 4). The detailed descriptions of the water physicochemical properties can be found in Table 2

The fraction of Bray-Curtis dissimilarity attributable to the stream_core averaged 40.6% at agricultural drainage ditch sites, which was significantly lower (*P* = 0.003) than that at mixed-use sites (60.3%). In contrast, stream_CRT’s contribution averaged 22.6% at agricultural drainage ditch sites, which was significantly greater (*P* = 0.004) than that at mixed-use sites (15.0%) (Fig. 3A). Moreover, the community compositional structure of the stream_core (perMANOVA F = 6.099, *P* = 0.001) and CRT (F = 3.188, *P* = 0.001) differed significantly between upstream land uses (supplementary Fig. S2B).

The seasonal patterns of stream_core and stream_CRT differed between the agricultural drainage ditch sites and mixed-use sites (Fig. 2A), although both contributed the least to the overall community variance at the beginning of the sampling season in 2017 and 2018, corresponding to the high relative water discharge and lower relative air temperature (below 10 °C) (supplementary Fig. S1). This was not observed in 2016 because of a late sampling start date (May 24, week 22) relative to 2017 (May 1, week 18) and 2018 (April 18, week 16). Through the sampling season, stream_core contribution at mixed-use sites increased quickly and reached a peak (> 70%) by the end of May of all 3 years (Fig. 2A), while at agricultural drainage ditch sites, it increased gradually and reached a relatively high level (> 60%) at a later time point (mid-July, week 28/29) in 2017 and 2018. The latter was not observed in 2016 because we were only able to collect five bi-weekly samples at agricultural drainage ditch sites that year due to dry conditions in the ditches. This observation suggested a lower restorative capacity (the ability of a community to return to its “normal” functioning after disturbance) of the aquatic microbiome at agricultural drainage ditch sites relative to mixed-use sites. At agricultural drainage ditch sites, the CRT contributed more to the overall community heterogeneity in 2016 and 2018 (*P* ≤ 0.01) than in 2017 (Fig. 2A); perhaps attributed to a relatively drier condition in 2016 and 2018 than in 2017 (supplementary Fig. S1). Such a yearly effect was not observed at mixed-use sites. The CRT contribution reached 51% during the week 34 (around August 20) of 2018 at agricultural drainage ditch sites (Fig. 2A).

The environmental factors potentially associated with the seasonal dynamics of stream_core and stream_CRT differed between agricultural drainage ditch sites and mixed-use sites according to the random forest algorithm (Fig. 2B). At agricultural drainage ditch sites, stream_core contribution was highly associated with dissolved oxygen, air temperature, dissolved organic C, daily averaged solar radiation, and water discharge, while that of stream_CRT was influenced more by conductivity, ammonia and ammonium, water discharge, and turbidity. In contrast, at the mixed-use sites, the seasonal variation of stream_core contribution was most affected by air or water temperature, turbidity, and pH, while the stream_CRT contribution varied with air or water temperature and daily average solar radiation.

We evaluated the stability of the bacterial community represented by core and CRT taxa. Not surprisingly, the stream_core exhibited higher stability than the stream_CRT in general. The stream_core at the agricultural drainage ditch sites were 2–3.5 folds less stable than that of other stream sites (*P* < 0.05) (Fig. 2C), but the stream_CRT communities displayed an opposite trend and showed higher stability at agricultural drainage ditch sites than the mixed use sites, although such differences were not significant (*P* > 0.05) (Fig. 2C).

### Core and CRT of agricultural drainage ditch sites are sensitive indicators of environmental change

To select more sensitive indicators for agricultural drainage ditch sites, we identified 551 ASVs (assigned to 50 genera) as core taxa and 4401 ASVs (194 genera) as CRT for ditch sites only, denoted as agri_core and agri_CRT (in contrast to stream_core and stream_CRT), respectively (supplementary Fig. S3A). The seasonal dynamics of the agri_core and agri_CRT exhibited the same temporal trends as the stream_core and stream_CRT (supplementary Fig. S3B).

Of the agri_core ASVs, those assigned to *Hydrogenophaga* (9 ASVs), *Hyphomicrobium* (3 ASVs), *Gallionella* (3 ASVs) were also identified as stream_core but were significantly more abundant at the agricultural drainage ditch sites than at other sites (Table 4). *Hyphomicrobium* and *Gallionella* ASVs were positively associated with rainfall, water discharge, dissolved oxygen and nitrate levels, and negatively correlated with ammonia and ammonium, total N and P, and TOC (Fig. 3A).

**Table 4:** Diversity and abundance of functional guilds identified as site-specific core taxa

Assigned genera	ASV numbers	Relative abundance (%)				***P*** value ^**c**^	Function or habitat ^**d**^
		Agri_ditches^**a**^	Agri_253^**b**^	Mixed	Forest		
***Agri_core identified as stream_core***							
*Hydrogenophaga*	12	0.416	0.129	0.101	0.061	0.037*	Hydrogenotrophic denitrification
*Hyphomicrobium*	12	0.094	0.017	0.033	0.010	0.034*	Denitrification
*Gallionella*	3	0.060	0.007	0.022	0.012	0.045*	Dark_iron_oxidation
***Agri_core not identified as stream_core***							
*Hydrogenophaga*	3	0.17	0.01	0.02	0.00	0.046*	Hydrogenotrophic denitrification
*Aquicella*	11	0.12	0.01	0.04	0.02	0.040*	Parasites; fecal indicator
*Polaromonas*	3	0.05	0.00	0.00	0.00	0.016*	Psychrophiles
*u114*	3	0.02	0.00	0.00	0.00	0.019*	Fish gut microbiome
*Mycoplana*	2	0.02	0.00	0.00	0.00	0.035*	Soil bacteria able to decompose aromatic compounds
*PSB-M-3*	2	0.02	0.00	0.00	0.00	0.050*	Phosphate-solubilizing bacteria
*Devosia*	2	0.02	0.00	0.00	0.00	0.026*	Nitrogen fixation; nitrate reduction

^a^ Agri_ditches, S18, S19, S20, and S21

^b^ Agri_253, site S253 is situated on a agriculturally-dominated stream

^c^ The *P* values represent the effects of land use classes based on linear mixed-effects modeling. The significance level is denoted at *, *P* ≤ 0.05

^d^ Citations are listed in Supplementary Table S1

We found 302 agri_core ASVs and 852 agri_CRT ASVs being identified as core or CRT only at agricultural ditch sites but not as core or CRT across stream sites (supplementary Fig. S3C). Of these site-specific agri_core ASVs, ASVs belonging to 7 genera were more abundant at agricultural drainage ditch sites than at all other sites (*P* < 0.05), and were affiliated with N metabolism, P solubilisation, or fecal indicators (Table 4). Of these, ASVs belonging to *Aquicella, Polaromonas, Mycoplana, and Devosia* were enriched in the spring, especially at the beginning of sampling season both in 2017 and 2018 (supplementary Fig. S4). These ASVs were also positively correlated with water discharge, dissolved oxygen, and nitrate concentrations (Fig. 3A). In contrast, PSB-M-3, a phosphate-solubilizing bacteria, was more abundant in 2016 and 2018 than in 2017 (supplementary Fig. S4), and was negatively correlated with rainfall and water discharge, and positively correlated with water nutrient (ammonia and ammonium, total N and P), C, and turbidity (Fig. 3A).

We highlight that agri_CRT ASVs affiliated with N metabolism, including N-fixation, assimilatory or dissimilatory nitrate reduction to ammonium, nitrification, and denitrification, were significantly and negatively correlated with rainfall, water discharge, nitrate, and ORP, and positively correlated with water N, P, C, and turbidity levels (Fig. 3A). In addition, these ASVs, especially those affiliated with N fixation, dissimilatory nitrate reduction and denitrification, were significantly more abundant at agricultural drainage ditch sites than at other sites (*P* < 0.05) and were highly enriched in 2016 and 2018 (dry year) relative to 2017 (wet year), and more abundant in summer than other seasons (Fig. 3B).

## Discussion

The core microbiome and CRT are important fractions of the microbial community, as emphasized in previous studies [29, 50–53]. In this study, we identified the common aquatic core microbiome and CRT across a suite of agriculturally-dominated waterways of varying stream order and use. The core taxa accounted for only 0.4% of total ASV numbers but contributed to the whole beta-diversity up to 86.9%, confirming the key role of the core microbiome in shaping the functioning of the microbiome in water [51, 53]. In contrast, the CRT represented a broader bacterial diversity than the core subcommunity, and was made up of taxa that can rapidly respond to environmental changes [29]. The CRT accounted for 5.2% of total ASV numbers, but their relative abundance accounted for 3.2 to 88% of the whole community and explained up to 51.6% of community dissimilarity. The high contribution of the stream_core and stream_CRT to the overall beta-diversity indicates that these two small subcommunities can effectively represent the dynamics of the aquatic microbiome as a whole. By focusing on a small set of ASVs (483 in stream_core and 6868 in stream_CRT vs. 131,809 in the overall bacterial community), we were able to effectively explore the temporal and spatial variations of the bacterial community diversity with a succinct reduction in computational complexity in comparison with analyzing the entire microbial community. Hence, core and CRT can be, from a pragmatic perspective, considered a robust tool for evaluating microbiome dynamics and drivers.

We demonstrated that core microbiome can be used to quantify the degree of the temporal variation of the bacterial community, therefore is a good indicator for assessing community stability across stream types [54, 55]. Stability refers to resistance to perturbations, resilience (the recovery speed after disturbance), and constancy (the degree of temporal stability) [45]. The constancy of the core taxa, as well as the combined core and CRT, was lower at the agricultural drainage ditch sites relative to all other stream types, suggesting the sensitivity and volatility of bacterial communities at agricultural ditch sites (Fig. 2C). This observation also suggested that the overall community stability was better represented by the temporal dynamics of the core microbiome than CRT. This was in line with Jiao et al. [53] who reported that the core microbiome drives the functional stability of microbiome in reforested ecosystems. Our results also suggested that the agricultural drainage ditch sites were less resilient to environmental perturbations. For example, in response to higher water discharge at the beginning of the sampling season, the contribution of the core microbiome to overall community variance at agricultural drainage ditch sites was as low as 20%, and it took a longer time to rise to 60% compared to that at mixed-use sites. The constancy and resilience of bacterial communities represented by core microbiome across sites demonstrated that this subcommunity well reflected ecosystem temporal stability across many different sized water courses under different primary uses.

To predict how microbial communities respond to environmental stressors and perturbations requires an understanding of environmental factors that influence its stability [51]. Changes in hydrology and water quality are important factors shaping the aquatic microbiome [1]. Our study demonstrated that both core microbiome and CRT contributed to the temporal variation of community diversity but corresponded to different environmental factors. Dissolved oxygen, air temperature, and water discharge were important factors associated with the core taxa contribution at the shallower agricultural drainage ditch sites where water physical and chemical conditions can be more volatile in response to environmental and anthropogenic influences [6] (Fig. 2B). We observed very low contributions of core taxa (< 20%) to overall community variance at the beginning and the end of the sampling seasons at agricultural drainage ditch sites, indicating an increase of community diversity in response to high water discharge (RU_DISM3S) during these periods (supplementary Fig. S1). Previous studies demonstrated that *E. coli* abundance and agro-chemical concentration can rapidly fluctuate based on precipitation and subsequent flow rates in such ditches due to their small size and proximity to agricultural fields [8, 56]. Smaller streams at our study sites can exhibit flashier hydrographs with peaks of flow in spring and fall, and intermittent stream flow during the summer months [33]. Indeed, our sampling started on May 1 in 2017 and on April 18 in 2018, during higher relative water flow conditions (supplementary Fig. S1), higher dissolved oxygen and nitrate levels; which were associated with bacterial denitrifiers in higher abundance (eg., *Janthinobacterium*, *Hyphomicrobium, Devosia)* [57].

The CRT were more sensitive to changes in system conditions than the core microbiome and significantly contributed to community temporal stability at agricultural drainage ditch sites. The high CRT contribution in the summer of 2016 and 2018 (drier years) at agricultural drainage ditch sites, was associated with higher levels of N and P loads and turbidity during this period, which perhaps, resulted from poor dilution due to the low flow induced by the dry condition. The observation that CRT represented the overall community heterogeneity better at agricultural drainage ditch sites than at mixed-use sites suggests that the watershed aquatic bacterial community was more responsive to environmental and anthropogenic perturbations in smaller ditch sites.

The core microbiome and CRT at agricultural drainage ditch sites (agri_core and agri_CRT) differed greatly from cross-site core and CRT (stream_core and stream_CRT). We found that more than 50% ASVs of agri_core were not identified as stream_core, which were significantly more abundant at agricultural drainage ditch sites relative to all other stream sites (supplementary Fig. S3D), indicating these ASVs could be good indicators specifically for agricultural drainage ditch ecosystem functions (e.g, dissipating and transforming agro-chemicals). Similar patterns were observed for members of agri_CRT relative to stream_CRT. This suggests that the identification of core and CRT from specific environments is necessary for detecting microbial indicators associated with different disturbances, such as rainfall events and surface runoff. The agri_core ASVs were enriched more at agricultural drainage ditch sites than at other stream sites (Table 4). These agri_core taxa could be used as environmental risk indicators specifically for agricultural drainage ditch sites because they provide specific ecological functions and services and respond differently to hydrology, water physicochemical properties, or weather events. For instance, the agri_core ASVs that were also identified as stream_core and belonging to *Hypomicrobium* (denitrifier) [57] and *Gallionella* (iron bacteria) [58] were abundant in the spring and fall seasons but rarely observed in summer, and were associated with high concentration of dissolved oxygen and water discharge (Fig. 3A, supplementary Fig. S4). The specific agri_core ASVs belonging to *Aquicella* (fecal indicator*), Polaromonas, Mycoplana,* and *Devosia,* were enriched in the spring, especially at the beginning of the 2017 and 2018 sampling seasons. As already mentioned, a phosphate-solubilizing bacteria, PSB-M-3, [59], was more abundant in 2016 and 2018 corresponding to higher P loadings in drier years than in a wet year (2017). Since P is usually mobilized in particulate matter in agricultural drainage ditches, the higher P loading may have originated from the internal P loadings associated with sediments [59, 60].

Because the shift in CRT at both agricultural drainage ditch sites and mixed-use sites was associated with ammonia and ammonium content (Fig. 2B), we hypothesized that this subcommunity is importantly involved in regulating N cycling. PICRUSt 2 analysis identified an array of bacterial taxa (2358 ASVs, accounting for 53.6% of total agri_CRT) affiliated with nitrate reduction, denitrification, and N-fixation, most of which showed higher abundance at agricultural drainage ditch sites relative to other stream sites and were more abundant in the summer of drier years, corresponding to high ammonia and ammonium levels of stream water. This is not surprising, as the applications of agrochemicals and organic fertilizers are usually scheduled in mid-late spring to early summer in this region, which may contaminate the drainage ditch streams through leaching or runoff. Previous studies have demonstrated that bacterioplankton community assemblages, in particular the CRT subcommunities, can be structured and shaped by both dispersal and recruitment (i.e., stochastic processes) and localized species sorting in response to environmental conditions and resources (i.e., deterministic processes) [28, 52, 61]. Strong correlations between water quality and functional guilds in agri_CRT suggest that these taxa are more likely shaped by environmental selection and can be used as risk indicators for environmental disturbances.

## Conclusion

Microbial activity governs, to a great degree, how an aquatic ecosystem cycles nutrients, dissipates pollutants, and provides the requisite food and habitat for wildlife. Thus, there is potential for the aquatic microbiome to be used as a holistic indicator of numerous aquatic ecosystem health endpoints. In this environmental observational study, we explored the core and CRT bacterial community dynamics in agricultural waterways including agricultural drainage ditches, which are first-line receptors of agricultural drainage and runoff. Some core findings are highlighted below:

1. Core and CRT were small fractions of the whole microbiome but well represented the overall community. They can be considered a holistic tool to explore the variations of the microbial community over time and space. They also appear useful in the context of indicators that express aquatic microbiome shifts associated with environmental changes. Moreover, the core and CRT approach will reduce computational complexities in relation to assessment of the entire microbiome community.
2. Changes in bacterial community structure in agricultural drainage ditch sites were predominantly associated with the CRT, while core microbiome well reflected ecosystem temporal stability across many different sized and used water courses.
3. The seasonal variations of the core taxa and CRT at agricultural drainage ditch sites were driven by hydrological conditions and water quality.
4. Many CRT show potential N cycling capabilities and are sensitive responders to high nutrient loading and lower water levels at agricultural drainage ditch sites.
5. Our study emphasized the importance of biomonitoring aquatic microbiome of agricultural drainage ditch streams for the assessment of stability and resilience of important microbial functionalities associated with processing anthropogenically derived agro-chemicals.

## Supplementary Information

**Additional file 1: Table S1.** Literatures for the functions and habits of bacterial genera presented in Table 4.

**Additional file 2: Supplementary Figure S1.** Seasonal dynamics of water physicochemical properties (A) and weather conditions (B).

**Additional file 3: Supplementary Figure S2.** (A) The relative abundance of the 20 most abundant genera in the stream_core (483 ASVs) (left) and stream_CRT (6868 ASVs) (right) subcommunities under different land use classes. Others_Core, core ASVs not belonging to the 20 most abundant genera in stream_core; non_core, ASVs not identified as core taxa; Others_CRT, CRT ASVs notbelonging to the 20 most abundant genera in stream_CRT; non_CRT, ASVs not identified as stream_CRT. (B) The beta-diversity of stream_core (top) and stream_CRT (bottom) under different land use classes, years, and sampling weeks, and their correlations with environmental conditions based on distance-based redundancy analysis (db-RDA).

**Additional file 4: Supplementary Figure S3.** Core (agri_core) and CRT (agri_CRT) at agricultural drainage ditch sites. (A) The relative abundances of agri_core and agri_CRT ASVs at the genus level. (B) Area plots represent the fractions of overall community Bray-Curtis dissimilarity (%) attributed to agri_core and agri_CRT over time in 2017 and 2018. The red area represents agri_core’s contribution; the green area represents agri_CRT’s contribution. (C) Venn diagram plots showing the numbers of ASVs that are unique or shared by stream_core and agri_core (top), and the numbers of ASVs that are unique or shared by stream_CRT and agri_CRT (bottom). (D) The relative abundances of ASVs in agri_core that were not identified as stream_core under the four land use classes.

**Additional file 5: Supplementary Figure S4.** Seasonal dynamics of bacterial functional guilds in core microbiome at agricultural drainage ditch sites (agri_core). (A) agri_core taxa identified as stream_core; (B) agri_core taxa that were not identified as stream_core. These functional guilds were more abundant at agricultural ditch sites than at other sampling sites.

## Data Availability

The raw paired-end 16S rRNA gene amplicon sequencing data have been deposited in the Sequence Read Archive (SRA), under the Bioproject accession PRJNA858176 (https://www.ncbi.nlm.nih.gov/bioproject/PRJNA858176), Biosample accessions SAMN29671977–2598.

## Abbreviations

CRT: Conditionally rare taxa
ASVs: Amplicon sequence variants
N: Nitrogen
C: Carbon
*P*: phosphorus
*stream_core*: the core microbiome across all study sites
*stream_CRT*: the conditionally rare taxa across all study sites
*agri_core*: the core microbiome at agricultural drainage ditches
*agri_CRT*: the conditionally rare taxa at agricultural drainage ditches
