# Supplementary material for: Core and conditionally rare taxa as indicators of agricultural drainage ditch and stream health and function
Source: BMC Microbiol. 2023 Mar 7;23:62. doi: 10.1186/s12866-023-02755-7 (PMC9990217; doi:10.1186/s12866-023-02755-7)
Supplement: Supplementary file 1 — Additional file 1: Table S1. Literatures for the functions and habits of bacterial genera presented in Table 4. [file 12866_2023_2755_MOESM1_ESM.pdf]

Supplementary Materials

Core and conditionally rare taxa as indicators of agricultural drainage ditch and stream health and function

Yichao Shi[1†], Izhar U.H. Khan[1], Devon Radford[1], Galen Guo[1], Mark Sunohara[1], Emilia Craiovan[1], David R. Lapen[1], Phillip Pham[1,2], Wen Chen[1,2†*]

[1] Ottawa Research and Development Centre, Agriculture and Agri-Food Canada, 960 Carling Avenue, Ottawa, Canada

[2] Department of Biology, University of Ottawa, Marie-Curie Private, Ottawa, ON K1N 9A7, Canada

[†] Y.S. and W.C contributed equally to this work.

*Correspondence: wen.chen@agr.gc.ca

Table S1. Literatures for the functions and habits of bacterial genera presented in Table 4.

Assigned genera	Function or habitat	Ref.
Agri_core identified as stream_core		
Hydrogenophaga	Hydrogenotrophic denitrification	(Xing et al. 2018)
Hyphomicrobium	Denitrification	(Shapleigh 2006)
Gallionella	Dark_iron_oxidation	(Hedrich et al. 2011)
Agri_core not identified as stream_core		
Hydrogenophaga	Hydrogenotrophic denitrification	(Xing et al. 2018)
Aquicella	Parasites; fecal indicator	(Saini and Gupta 2021)
Polaromonas	Psychrophiles	(Darcy et al. 2011)
u114	Fish gut microbiome	(Liu et al. 2018)
Mycoplana	Soil bacteria able to decompose aromatic compounds	(Urakami et al. 1990)
PSB-M-3	Phosphate-solubilizing bacteria	(Wang et al. 2018)
Devosia	Nitrogen fixation; nitrate reduction	(Jurburg et al. 2017)

Reference lists:

Darcy, J.L., Lynch, R.C., King, A.J., Robeson, M.S. and Schmidt, S.K. (2011) Global Distribution of Polaromonas Phylotypes - Evidence for a Highly Successful Dispersal Capacity. Plos One 6(8), e23742.

Hedrich, S., Schlömann, M. and Johnson, D.B. (2011) The iron-oxidizing proteobacteria. Microbiology 157(6), 1551-1564.

Jurburg, S.D., Nunes, I., Stegen, J.C., Le Roux, X., Priemé, A., Sørensen, S.J. and Salles, J.F. (2017) Autogenic succession and deterministic recovery following disturbance in soil bacterial communities. Sci. Rep. 7(1), 45691.

Liu, J., Li, C., Jing, J., Zhao, P., Luo, Z., Cao, M., Ma, Z., Jia, T. and Chai, B. (2018) Ecological patterns and adaptability of bacterial communities in alkaline copper mine drainage. Water Res. 133, 99-109.

Saini, N. and Gupta, R.S. (2021) A robust phylogenetic framework for members of the order Legionellales and its main genera (Legionella, Aquicella, Coxiella and Rickettsiella) based on phylogenomic analyses and identification of molecular markers demarcating different clades. Antonie Van Leeuwenhoek 114(7), 957-982.

Shapleigh, J.P. (2006) The denitrifying prokaryotes. The prokaryotes 2, 769-792.

Urakami, T., Oyanagi, H., Araki, H., Suzuki, K.-I. and Komagata, K. (1990) Recharacterization and Emended Description of the Genus Mycoplana and Description of Two New Species, Mycoplana ramosa and Mycoplana segnis. Int. J. Syst. Evol. Microbiol. 40(4), 434-442.

Xing, W., Li, J., Li, P., Wang, C., Cao, Y., Li, D., Yang, Y., Zhou, J. and Zuo, J. (2018) Effects of residual organics in municipal wastewater on hydrogenotrophic denitrifying microbial communities. J Environ Sci (China) 65, 262-270.

Wang, C., Liu, Z., Zhang, Y., Liu, B., Zhou, Q., Zeng, L., He, F. and Wu, Z. (2018) Synergistic removal effect of P in sediment of all fractions by combining the modified bentonite granules and submerged macrophyte. Sci. Total Environ. 626, 458-467.
