# Supplementary material for: Core and conditionally rare taxa as indicators of agricultural drainage ditch and stream health and function
Source: BMC Microbiol. 2023 Mar 7;23:62. doi: 10.1186/s12866-023-02755-7 (PMC9990217; doi:10.1186/s12866-023-02755-7)
Supplement: Supplementary file 2 — Additional file 2: Supplementary Figure S1. Seasonal dynamics of water physicochemical properties (A) and weather conditions (B). [file 12866_2023_2755_MOESM2_ESM.pdf]

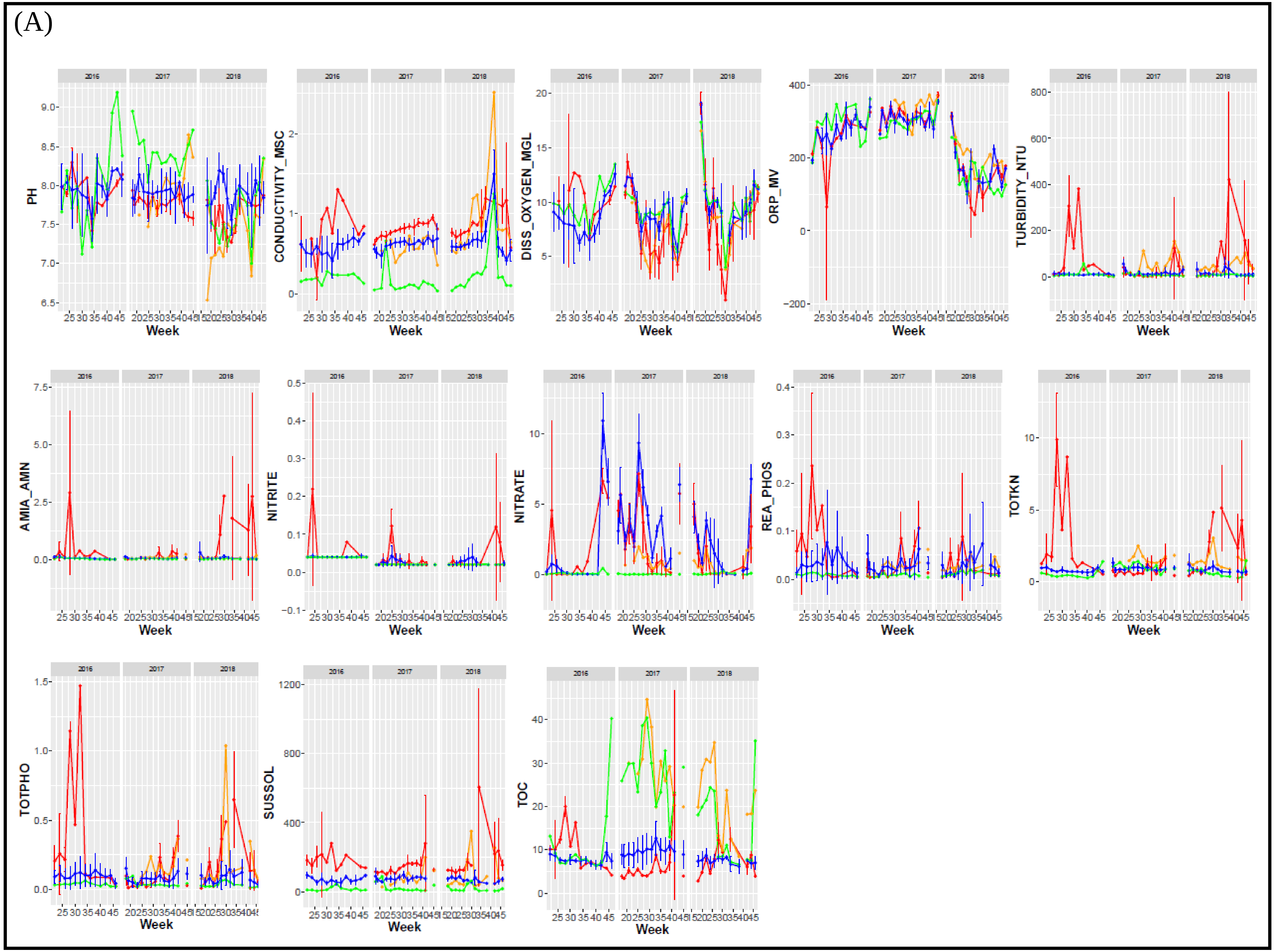

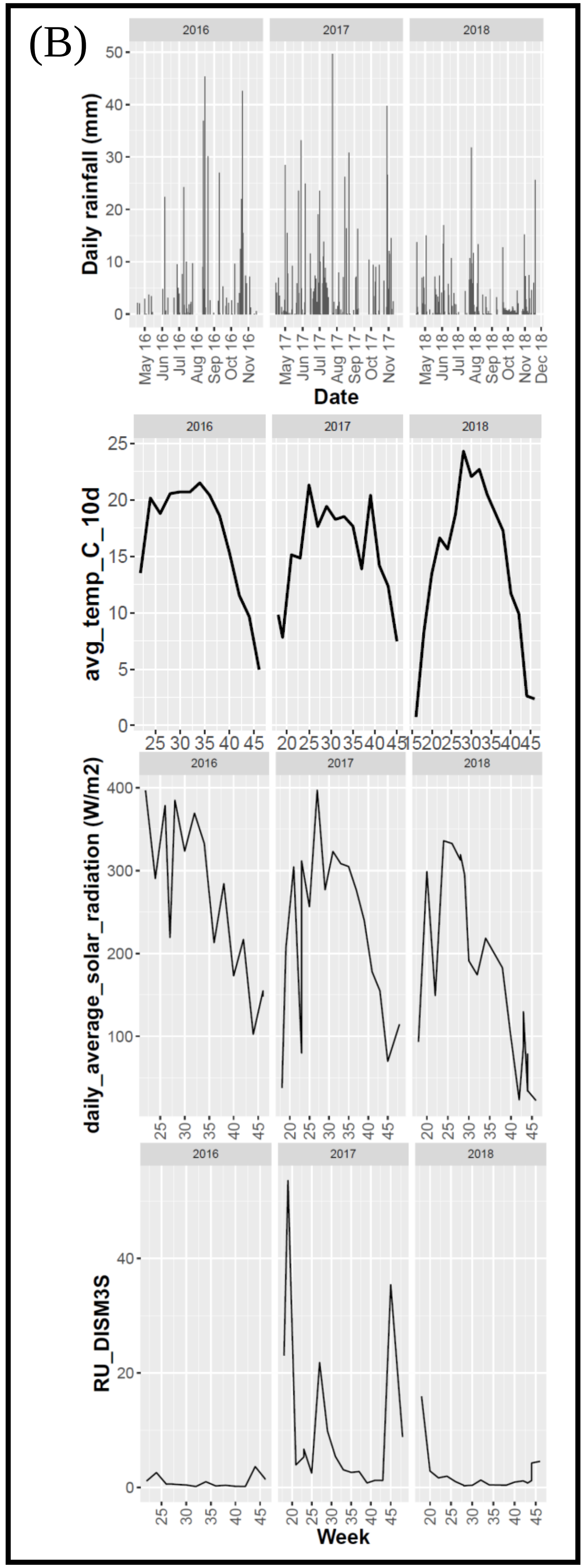

Supplementary Figure S1. Seasonal dynamics of water physicochemical properties (A) and weather conditions (B).
