# Supplementary material for: Core and conditionally rare taxa as indicators of agricultural drainage ditch and stream health and function
Source: BMC Microbiol. 2023 Mar 7;23:62. doi: 10.1186/s12866-023-02755-7 (PMC9990217; doi:10.1186/s12866-023-02755-7)
Supplement: Supplementary file 3 — Additional file 3: Supplementary Figure S2. (A) The relative abundance of the 20 most abundant genera in the stream_core (483 ASVs) (left) and stream_CRT (6868 ASVs) (right) subcommunities under different land use classes. Others_Core, core ASVs not belonging to the 20 most abundant genera in stream_core; non_core, ASVs not identified as core taxa; Others_CRT, CRT ASVs notbelonging to the 20 most abundant genera in stream_CRT; non_CRT, ASVs not identified as stream_CRT. (B) The beta-diversity of stream_core (top) and stream_CRT (bottom) under different land use classes, years, and sampling weeks, and their correlations with environmental conditions based on distance-based redundancy analysis (db-RDA). [file 12866_2023_2755_MOESM3_ESM.pdf]

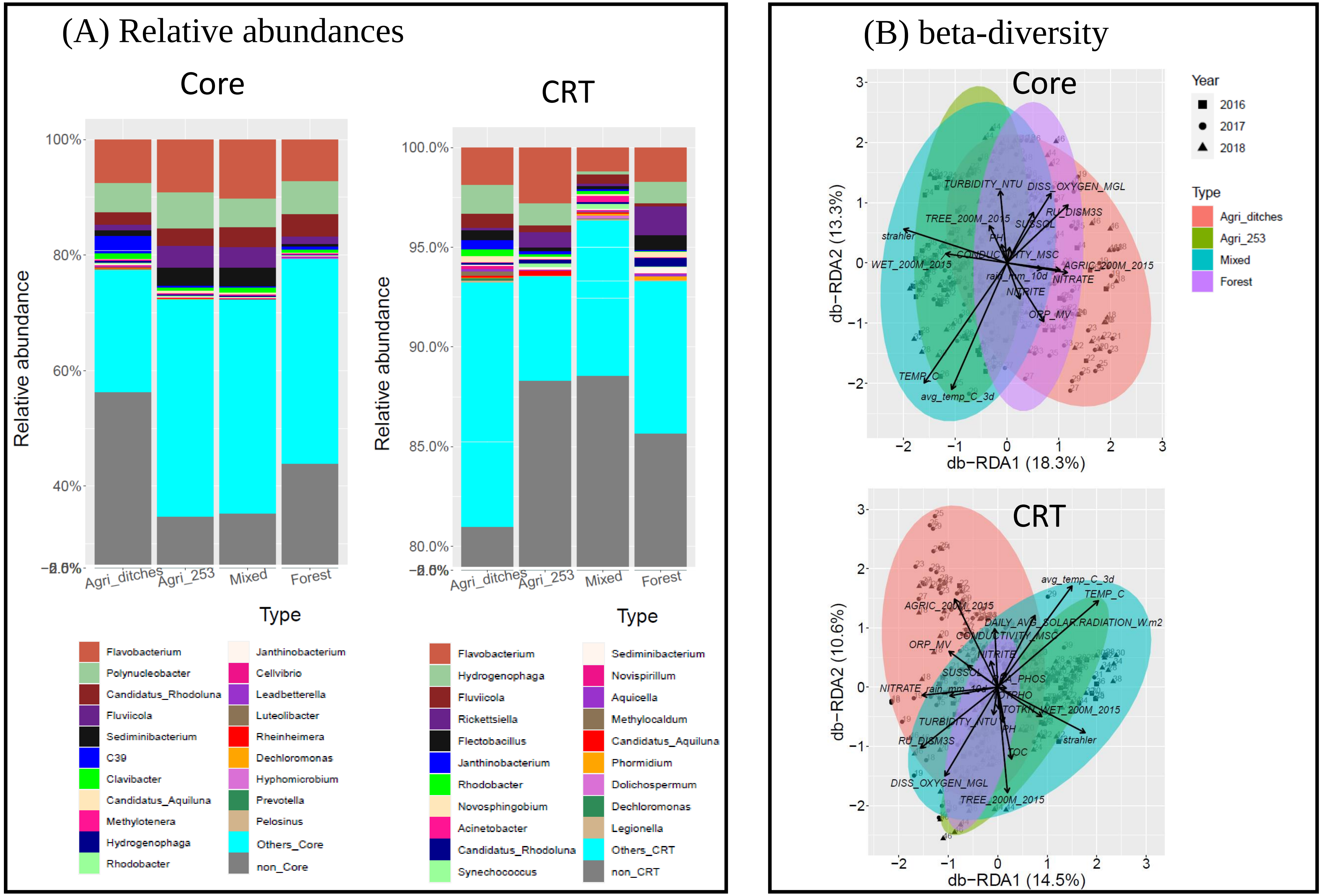

Supplementary Figure S2. (A) The relative abundance of the 20 most abundant genera in the stream_core (483 ASVs) (left) and stream_CRT (6868 ASVs) (right) subcommunities under different land use classes. Others_Core, core ASVs not belonging to the 20 most abundant genera in stream_core; non_core, ASVs not identified as core taxa; Others_CRT, CRT ASVs not belonging to the 20 most abundant genera in stream_CRT; non_CRT, ASVs not identified as stream_CRT. (B) The beta-diversity of stream_core (top) and stream_CRT (bottom) under different land use classes, year, and sampling weeks, and their correlations with environmental conditions based on distance-based redundancy analysis (db-RDA).
