# Supplementary material for: Core and conditionally rare taxa as indicators of agricultural drainage ditch and stream health and function
Source: BMC Microbiol. 2023 Mar 7;23:62. doi: 10.1186/s12866-023-02755-7 (PMC9990217; doi:10.1186/s12866-023-02755-7)
Supplement: Supplementary file 4 — Additional file 4: Supplementary Figure S3. Core (agri_core) and CRT (agri_CRT) at agricultural drainage ditch sites. (A) The relative abundances of agri_core and agri_CRT ASVs at the genus level. (B) Area plots represent the fractions of overall community Bray-Curtis dissimilarity (%) attributed to agri_core and agri_CRT over time in 2017 and 2018. The red area represents agri_core’s contribution; the green area represents agri_CRT’s contribution. (C) Venn diagram plots showing the numbers of ASVs that are unique or shared by stream_core and agri_core (top), and the numbers of ASVs that are unique or shared by stream_CRT and agri_CRT (bottom). (D) The relative abundances of ASVs in agri_core that were not identified as stream_core under the four land use classes. [file 12866_2023_2755_MOESM4_ESM.pdf]

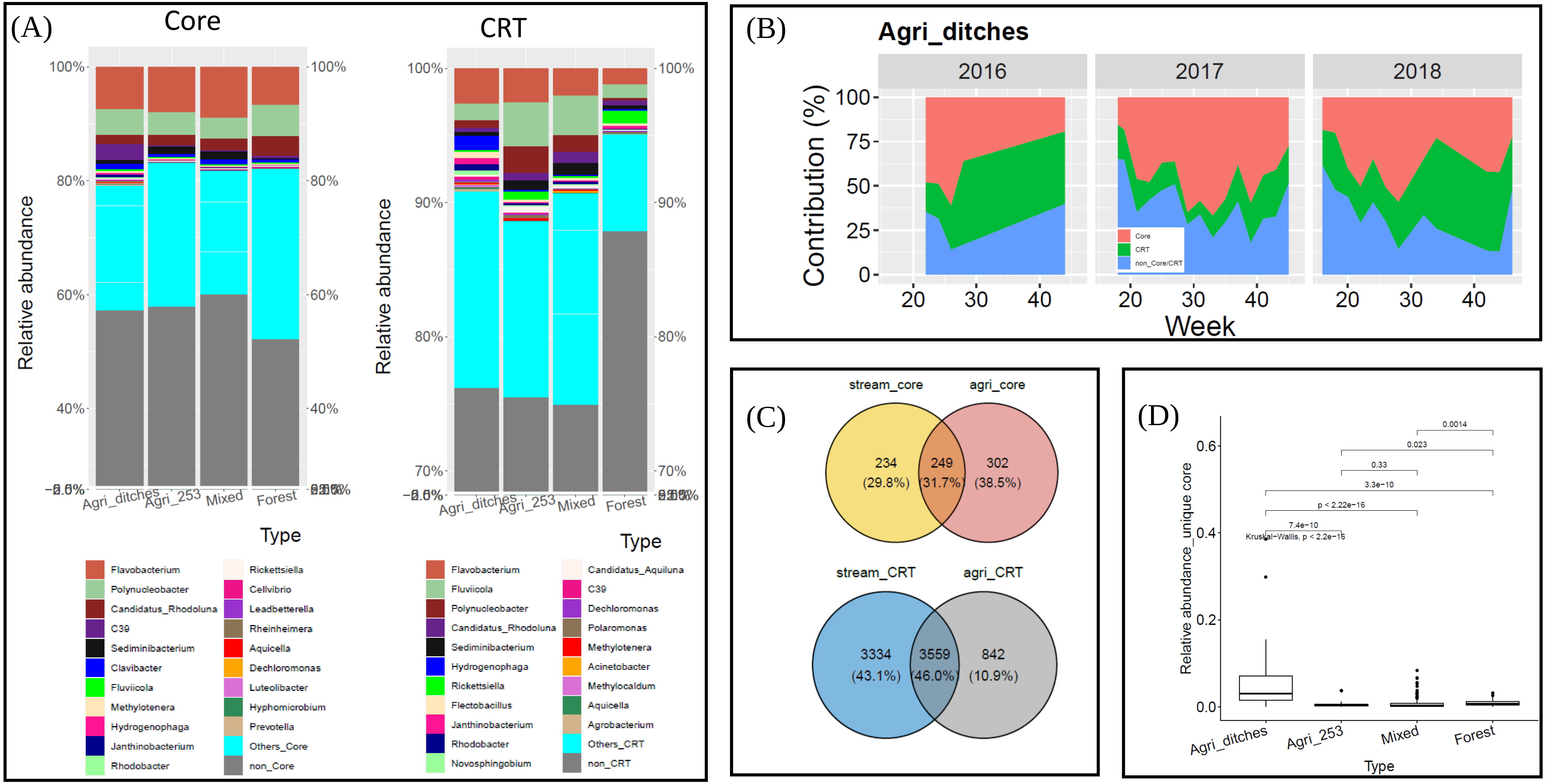

Supplementary Figure S3. Core (agri_core) and CRT (agri_CRT) at agricultural drainage ditch sites. (A) The relative abundances of agri_core and agri_CRT ASVs at the genus level. (B) Area plots represent the fraction of overall community Bray-Curtis dissimilarity (%) attributed to agri_core and agri_CRT over time in 2017 and 2018. The red area represents agri_core's contribution; the green area represents agri_CRT's contribution. (C) Venn diagram plot showing the number of ASVs that are unique or shared by the stream_core and agri_core (top), and the number of ASVs that are unique or shared by the stream_CRT and agri_CRT (bottom). (D) The relative abundance of ASVs in agri_core that were not identified as stream_core under the four land use classes.
