# Supplementary figures and images for: Core and conditionally rare taxa as indicators of agricultural drainage ditch and stream health and function
Source: BMC Microbiol. 2023 Mar 7;23:62. doi: 10.1186/s12866-023-02755-7 (PMC9990217; doi:10.1186/s12866-023-02755-7)

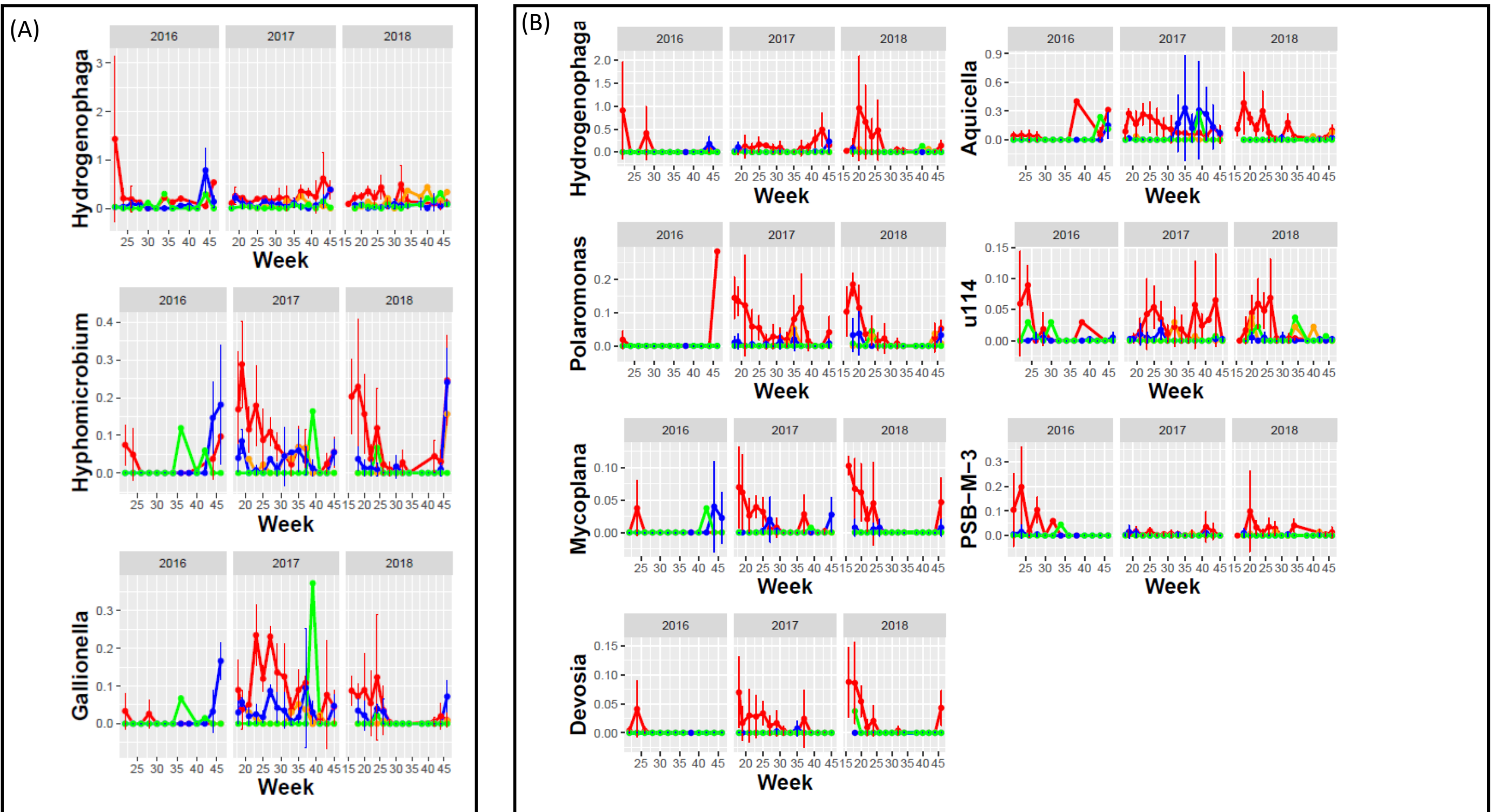

Supplement: Supplementary file 5 — Additional file 5: Supplementary Figure S4. Seasonal dynamics of bacterial functional guilds in core microbiome at agricultural drainage ditch sites (agri_core). (A) agri_core taxa identified as stream_core; (B) agri_core taxa that were not identified as stream_core. These functional guilds were more abundant at agricultural ditch sites than at other sampling sites. [file 12866_2023_2755_MOESM5_ESM.pdf]
